# 2-[(*E*)-(2-Morpholinoeth­yl)iminiometh­yl]-4-nitro-1-oxocyclo­hexa­dienide

**DOI:** 10.1107/S1600536809026191

**Published:** 2009-07-11

**Authors:** Yelda Bingöl Alpaslan, Hasan Tanak, Erbil Ağar, Ferda Erşahin

**Affiliations:** a Department of Physics, Faculty of Arts & Science, Ondokuz Mayıs University, TR-55139 Kurupelit–Samsun, Turkey; b Department of Chemistry, Faculty of Arts & Science, Ondokuz Mayıs University, 55139 Samsun, Turkey

## Abstract

The mol­ecule of the title compound, C_13_H_17_N_3_O_4_, exists as a zwitterion, with the H atom of the phenol group being transferred to the imine N atom. The C=O, C_Ar_—C_Ar_ and C—N bond lengths are in agreement with the oxocyclo­hexa­dienide–iminium zwitterionic form. A strong intra­molecular N^+^—H⋯O hydrogen bond generates an *S*(6) ring motif. The morpholine ring adopts a chair conformation. In the crystal, mol­ecules are linked into centrosymmetric dimers by inter­molecular N—H⋯O hydrogen bonds. In addition, C—H⋯O hydrogen bonds and very weak C—H⋯π inter­actions are observed.

## Related literature

For general background, photochromic and thermochromic characteristics of Schiff base compounds, see: Calligaris *et al.* (1972 ▶); Cohen *et al.* (1964 ▶); Hadjoudis *et al.* (1987 ▶); Karabıyık *et al.* (2008 ▶). For related structures, see: Butt *et al.* (1987 ▶); Petek *et al.* (2006 ▶); Krygowski & Stepien (2005 ▶); Santos-Contreras *et al.* (2009 ▶). For graph-set analysis of hydrogen bonds, see: Bernstein *et al.* (1995 ▶).
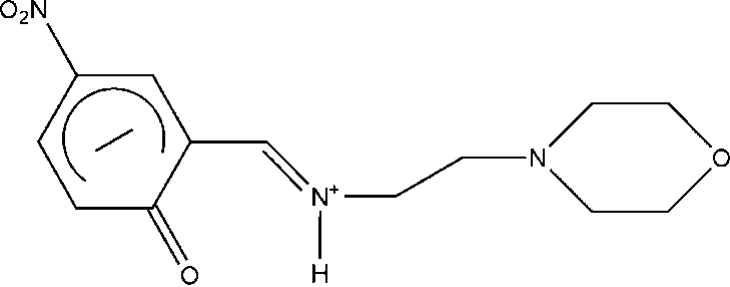


## Experimental

### 

#### Crystal data

C_13_H_17_N_3_O_4_
*M* *_r_* = 279.30
Triclinic, 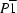

*a* = 5.3520 (4) Å
*b* = 10.8972 (9) Å
*c* = 12.4537 (9) Å
α = 102.329 (7)°
β = 97.143 (6)°
γ = 104.173 (9)°
*V* = 675.91 (10) Å^3^
*Z* = 2
Mo *K*α radiation
μ = 0.10 mm^−1^
*T* = 296 K
0.75 × 0.70 × 0.40 mm

#### Data collection

Stoe IPDSII diffractometer
Absorption correction: none
11340 measured reflections
3094 independent reflections
2664 reflections with *I* > 2σ(*I*)
*R*_int_ = 0.039

#### Refinement

*R*[*F* ^2^ > 2σ(*F* ^2^)] = 0.040
*wR*(*F* ^2^) = 0.110
*S* = 1.06
3094 reflections
186 parameters
H atoms treated by a mixture of independent and constrained refinement
Δρ_max_ = 0.26 e Å^−3^
Δρ_min_ = −0.17 e Å^−3^

### 

Data collection: *X-AREA* (Stoe & Cie, 2002 ▶); cell refinement: *X-AREA*; data reduction: *X-RED32* (Stoe & Cie, 2002 ▶); program(s) used to solve structure: *SHELXS97* (Sheldrick, 2008 ▶); program(s) used to refine structure: *SHELXL97* (Sheldrick, 2008 ▶); molecular graphics: *ORTEP-3 for Windows* (Farrugia, 1997 ▶); software used to prepare material for publication: *WinGX* (Farrugia, 1999 ▶).

## Supplementary Material

Crystal structure: contains datablocks I, global. DOI: 10.1107/S1600536809026191/ci2842sup1.cif

Structure factors: contains datablocks I. DOI: 10.1107/S1600536809026191/ci2842Isup2.hkl

Additional supplementary materials:  crystallographic information; 3D view; checkCIF report

## Figures and Tables

**Table 1:** Hydrogen-bond geometry (Å, °)

*D*—H⋯*A*	*D*—H	H⋯*A*	*D*⋯*A*	*D*—H⋯*A*
N2—H1⋯O1	0.89 (2)	1.99 (2)	2.6760 (14)	133 (2)
N2—H1⋯O1^i^	0.89 (2)	2.24 (2)	2.9587 (14)	138 (2)
C4—H4⋯O4^ii^	0.93	2.47	3.3547 (16)	160
C7—H7⋯O3^iii^	0.93	2.43	3.3020 (15)	157
C13—H13*B*⋯*Cg*2^iv^	0.97	2.99	3.9254	162

Symmetry codes: (i) 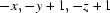; (ii) 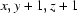; (iii) 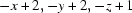; (iv) 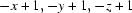. *Cg*2 is the centroid of the C1–C6 ring.

**Fig. 1.**
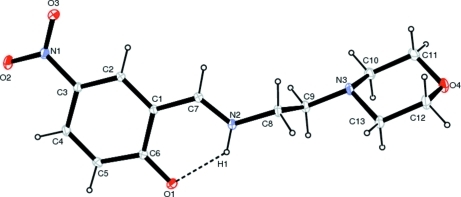
The molecular structure of (I), showing the atom-numbering scheme. Displacement ellipsoids are drawn at the 50% probability level and H atoms are shown as small spheres of arbitrary radii.

**Fig. 2.**
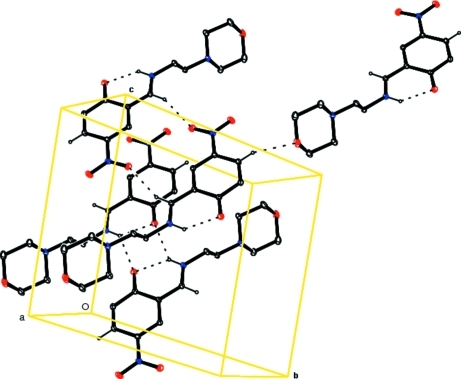
A packing diagram for (I), showing O—H···O hydrogen-bonded (dashed lines) dimers. H atoms not involved in hydrogen bonding have been omitted for clarity.

## supplementary crystallographic information

### Comment

Schiff bases have been extensively used as ligands in the field of coordination chemistry (Calligaris *et al.*, 1972). Schiff base compounds can be classified by their photochromic and thermochromic characteristics (Cohen *et al.* 1964). These properties result from proton transfer from the hydroxyl O atom to the imine N atom (Hadjoudis *et al.*, 1987). Schiff bases exhibit two well-known tautomeric forms viz. OH and NH tautomers, and they also exist in zwitterionic form (Karabıyık *et al.*, 2008). Our investigations show that compund (I) exists in a zwitterionic form.

The molecular structure of (I) is shown in Fig.1. The C1—C7 [1.4241 (15) Å], C7═N2 [1.2894 (15) Å] and N2—C8 [1.4615 (14) Å] bond lengths agree with the corresponding distances in (E)-2-methoxy-6-[(2- morpholinoethylimino)methyl]phenolate [1.425 (2), 1.287 (2) and 1.464 (2) Å; Petek *et al.*, 2006]. The bonds lengths in the C1-C6 benzene ring show clear alternation in the delocalized C2-C5 portion. The nitro group is tilted out of the mean plane of adjacent ring by 7.02 (3)°, whereas the C3—N1 distance of 1.4398 (15) Å is in the characteristic range suggesting limited conjugation with the ring. Thus, the whole geometry is in the agreement with the predominace of the oxocyclohexadienide-iminum zwitterion bonding scheme (see scheme) (Krygowski & Stepien, 2005; Santos-Contreras *et al.*, 2009), in close agreement with the reported configurations of p-nitrophenolates of alkali metal cations (Butt *et al.*, 1987). The morpholine ring adopts a chair conformation. An intramolecular N—H···O hydrogen bond generates an S(6) ring motif (Bernstein *et al.*, 1995).

In the crystal structure of (I), the molecules form centrosymmetric dimers connected by intermolecular N—H···O hydrogen bonds (Fig. 2). In addition, C—H···O hydrogen bonds and very weak C—H···π interactions between C13-H13B group and C1-C6 benzene ring are observed (Table 1).

### Experimental

2-Hydroxy-5-nitrobenzaldehyde (0.0535 g, 0.32 mmol) in ethanol (20 ml) was added to 2-morpholinoethylamine (0.0417 g, 0.32 mmol) in ethanol (20 ml) and the reaction mixture was stirred for 1 h under reflux. Single crystals of the title compound suitable for X-ray analysis were obtained by slow evaporation of an ethanol solution (yield % 65; m.p. 435-438 K).

### Refinement

Atom H1 was located in a difference map and refined freely. All other H atoms were placed in calculated positions and constrained to ride on their parent atoms, with C-H = 0.93–0.97 Å and *U*_iso_(H) = 1.2 *U*_eq_(C).

### Crystal data

**Table:**

C_13_H_17_N_3_O_4_	*Z* = 2
*M**_r_* = 279.30	*F*(000) = 296
Triclinic, *P*1	*D*_x_ = 1.372 Mg m^−^^3^
Hall symbol: -P 1	Mo *K*α radiation, λ = 0.71073 Å
*a* = 5.3520 (4) Å	Cell parameters from 23053 reflections
*b* = 10.8972 (9) Å	θ = 1.7–28.0°
*c* = 12.4537 (9) Å	µ = 0.10 mm^−^^1^
α = 102.329 (7)°	*T* = 296 K
β = 97.143 (6)°	Prism, orange
γ = 104.173 (9)°	0.75 × 0.70 × 0.40 mm
*V* = 675.91 (10) Å^3^	

### Data collection

**Table:**

Stoe IPDSII diffractometer	2664 reflections with *I* > 2σ(*I*)
Radiation source: fine-focus sealed tube	*R*_int_ = 0.039
graphite	θ_max_ = 27.6°, θ_min_ = 1.7°
Detector resolution: 6.67 pixels mm^-1^	*h* = −6→6
ω scans	*k* = −14→14
11340 measured reflections	*l* = −16→16
3094 independent reflections	

### Refinement

**Table:**

Refinement on *F*^2^	Secondary atom site location: difference Fourier map
Least-squares matrix: full	Hydrogen site location: inferred from neighbouring sites
*R*[*F*^2^ > 2σ(*F*^2^)] = 0.040	H atoms treated by a mixture of independent and constrained refinement
*wR*(*F*^2^) = 0.110	*w* = 1/[σ^2^(*F*_o_^2^) + (0.0512*P*)^2^ + 0.1309*P*] where *P* = (*F*_o_^2^ + 2*F*_c_^2^)/3
*S* = 1.06	(Δ/σ)_max_ = 0.001
3094 reflections	Δρ_max_ = 0.26 e Å^−^^3^
186 parameters	Δρ_min_ = −0.17 e Å^−^^3^
0 restraints	Extinction correction: *SHELXL97* (Sheldrick, 2008), Fc^*^=kFc[1+0.001xFc^2^λ^3^/sin(2θ)]^-1/4^
Primary atom site location: structure-invariant direct methods	Extinction coefficient: 0.077 (7)

### Special details

**Table:**

Geometry. All esds (except the esd in the dihedral angle between two l.s. planes) are estimated using the full covariance matrix. The cell esds are taken into account individually in the estimation of esds in distances, angles and torsion angles; correlations between esds in cell parameters are only used when they are defined by crystal symmetry. An approximate (isotropic) treatment of cell esds is used for estimating esds involving l.s. planes.
Refinement. Refinement of F^2^ against ALL reflections. The weighted R-factor wR and goodness of fit S are based on F^2^, conventional R-factors R are based on F, with F set to zero for negative F^2^. The threshold expression of F^2^ > 2sigma(F^2^) is used only for calculating R-factors(gt) etc. and is not relevant to the choice of reflections for refinement. R-factors based on F^2^ are statistically about twice as large as those based on F, and R- factors based on ALL data will be even larger.

### Fractional atomic coordinates and isotropic or equivalent isotropic displacement parameters (Å^2^)

**Table:**

	*x*	*y*	*z*	*U*_iso_*/*U*_eq_	
C1	0.4611 (2)	0.77887 (11)	0.53094 (9)	0.0335 (2)	
C2	0.6439 (2)	0.90243 (11)	0.56526 (9)	0.0341 (2)	
H2	0.7896	0.9204	0.5315	0.041*	
C3	0.6067 (2)	0.99713 (10)	0.64924 (9)	0.0348 (3)	
C4	0.3899 (3)	0.97257 (12)	0.70256 (10)	0.0396 (3)	
H4	0.3692	1.0377	0.7597	0.048*	
C5	0.2112 (3)	0.85348 (12)	0.67033 (11)	0.0426 (3)	
H5	0.0698	0.8382	0.7069	0.051*	
C6	0.2318 (2)	0.74989 (11)	0.58183 (10)	0.0363 (3)	
C7	0.5074 (2)	0.68324 (11)	0.44412 (9)	0.0358 (3)	
H7	0.6566	0.7074	0.4138	0.043*	
C8	0.4146 (3)	0.46932 (12)	0.31724 (10)	0.0430 (3)	
H8A	0.3937	0.3878	0.3393	0.052*	
H8B	0.5956	0.5004	0.3093	0.052*	
C9	0.2370 (3)	0.44450 (12)	0.20590 (10)	0.0437 (3)	
H9A	0.0572	0.4050	0.2113	0.052*	
H9B	0.2453	0.5268	0.1865	0.052*	
C10	0.2038 (3)	0.36092 (15)	0.00679 (11)	0.0513 (3)	
H10A	0.2527	0.4500	−0.0009	0.062*	
H10B	0.0140	0.3307	−0.0047	0.062*	
C11	0.3025 (3)	0.27445 (17)	−0.07913 (12)	0.0640 (4)	
H11A	0.2304	0.2783	−0.1534	0.077*	
H11B	0.4920	0.3062	−0.0681	0.077*	
C12	0.3364 (4)	0.13651 (16)	0.03730 (15)	0.0665 (5)	
H12A	0.5264	0.1645	0.0487	0.080*	
H12B	0.2834	0.0467	0.0429	0.080*	
C13	0.2450 (3)	0.22200 (13)	0.12725 (12)	0.0504 (3)	
H13A	0.0561	0.1904	0.1194	0.060*	
H13B	0.3244	0.2177	0.2002	0.060*	
N1	0.7884 (2)	1.12600 (10)	0.68031 (9)	0.0410 (3)	
N2	0.3567 (2)	0.56554 (9)	0.40467 (8)	0.0378 (2)	
N3	0.3171 (2)	0.35764 (10)	0.11888 (8)	0.0422 (3)	
O1	0.06162 (19)	0.64042 (9)	0.55030 (9)	0.0513 (3)	
O2	0.7384 (2)	1.21314 (9)	0.74731 (9)	0.0600 (3)	
O3	0.9854 (2)	1.14568 (9)	0.63771 (9)	0.0549 (3)	
O4	0.2302 (3)	0.14272 (12)	−0.07115 (9)	0.0727 (4)	
H1	0.214 (4)	0.5433 (18)	0.4335 (15)	0.065 (5)*	

### Atomic displacement parameters (Å^2^)

**Table:**

	*U*^11^	*U*^22^	*U*^33^	*U*^12^	*U*^13^	*U*^23^
C1	0.0382 (6)	0.0302 (5)	0.0305 (5)	0.0089 (4)	0.0086 (4)	0.0041 (4)
C2	0.0370 (6)	0.0324 (5)	0.0316 (5)	0.0083 (4)	0.0096 (4)	0.0055 (4)
C3	0.0410 (6)	0.0286 (5)	0.0310 (5)	0.0073 (4)	0.0048 (4)	0.0035 (4)
C4	0.0493 (7)	0.0363 (6)	0.0335 (6)	0.0162 (5)	0.0120 (5)	0.0020 (4)
C5	0.0450 (7)	0.0419 (6)	0.0422 (6)	0.0125 (5)	0.0198 (5)	0.0065 (5)
C6	0.0388 (6)	0.0319 (5)	0.0372 (6)	0.0085 (4)	0.0102 (5)	0.0063 (4)
C7	0.0398 (6)	0.0336 (5)	0.0323 (5)	0.0088 (5)	0.0098 (4)	0.0049 (4)
C8	0.0508 (7)	0.0361 (6)	0.0374 (6)	0.0151 (5)	0.0086 (5)	−0.0038 (5)
C9	0.0545 (7)	0.0391 (6)	0.0375 (6)	0.0195 (5)	0.0105 (5)	0.0008 (5)
C10	0.0532 (8)	0.0582 (8)	0.0358 (7)	0.0119 (6)	0.0063 (6)	0.0030 (6)
C11	0.0646 (10)	0.0736 (10)	0.0367 (7)	0.0046 (8)	0.0132 (6)	−0.0071 (7)
C12	0.0776 (11)	0.0501 (8)	0.0648 (10)	0.0231 (8)	0.0203 (8)	−0.0105 (7)
C13	0.0625 (9)	0.0409 (7)	0.0457 (7)	0.0180 (6)	0.0147 (6)	−0.0003 (5)
N1	0.0491 (6)	0.0311 (5)	0.0371 (5)	0.0070 (4)	0.0045 (4)	0.0030 (4)
N2	0.0443 (6)	0.0317 (5)	0.0331 (5)	0.0083 (4)	0.0105 (4)	−0.0002 (4)
N3	0.0497 (6)	0.0406 (5)	0.0324 (5)	0.0146 (5)	0.0088 (4)	−0.0020 (4)
O1	0.0483 (5)	0.0358 (5)	0.0617 (6)	−0.0002 (4)	0.0219 (4)	0.0025 (4)
O2	0.0719 (7)	0.0337 (5)	0.0622 (6)	0.0106 (4)	0.0133 (5)	−0.0092 (4)
O3	0.0556 (6)	0.0417 (5)	0.0564 (6)	−0.0031 (4)	0.0169 (5)	0.0047 (4)
O4	0.0816 (8)	0.0612 (7)	0.0526 (6)	0.0055 (6)	0.0197 (6)	−0.0207 (5)

### Geometric parameters (Å, °)

**Table:**

C1—C2	1.3994 (16)	C9—H9A	0.97
C1—C7	1.4241 (15)	C9—H9B	0.97
C1—C6	1.4478 (16)	C10—N3	1.4625 (17)
C2—C3	1.3748 (15)	C10—C11	1.505 (2)
C2—H2	0.93	C10—H10A	0.97
C3—C4	1.4051 (17)	C10—H10B	0.97
C3—N1	1.4398 (15)	C11—O4	1.421 (2)
C4—C5	1.3543 (18)	C11—H11A	0.97
C4—H4	0.93	C11—H11B	0.97
C5—C6	1.4357 (16)	C12—O4	1.421 (2)
C5—H5	0.93	C12—C13	1.5086 (19)
C6—O1	1.2594 (14)	C12—H12A	0.97
C7—N2	1.2894 (15)	C12—H12B	0.97
C7—H7	0.93	C13—N3	1.4632 (18)
C8—N2	1.4615 (14)	C13—H13A	0.97
C8—C9	1.5117 (18)	C13—H13B	0.97
C8—H8A	0.97	N1—O2	1.2293 (14)
C8—H8B	0.97	N1—O3	1.2315 (14)
C9—N3	1.4596 (15)	N2—H1	0.887 (19)
			
C2—C1—C7	117.84 (10)	N3—C10—H10A	109.8
C2—C1—C6	120.88 (10)	C11—C10—H10A	109.8
C7—C1—C6	121.27 (10)	N3—C10—H10B	109.8
C3—C2—C1	119.54 (10)	C11—C10—H10B	109.8
C3—C2—H2	120.2	H10A—C10—H10B	108.2
C1—C2—H2	120.2	O4—C11—C10	111.17 (13)
C2—C3—C4	121.54 (10)	O4—C11—H11A	109.4
C2—C3—N1	119.24 (10)	C10—C11—H11A	109.4
C4—C3—N1	119.18 (10)	O4—C11—H11B	109.4
C5—C4—C3	119.65 (11)	C10—C11—H11B	109.4
C5—C4—H4	120.2	H11A—C11—H11B	108.0
C3—C4—H4	120.2	O4—C12—C13	111.48 (14)
C4—C5—C6	122.50 (11)	O4—C12—H12A	109.3
C4—C5—H5	118.8	C13—C12—H12A	109.3
C6—C5—H5	118.8	O4—C12—H12B	109.3
O1—C6—C5	122.23 (11)	C13—C12—H12B	109.3
O1—C6—C1	121.91 (10)	H12A—C12—H12B	108.0
C5—C6—C1	115.86 (10)	N3—C13—C12	110.35 (13)
N2—C7—C1	124.56 (11)	N3—C13—H13A	109.6
N2—C7—H7	117.7	C12—C13—H13A	109.6
C1—C7—H7	117.7	N3—C13—H13B	109.6
N2—C8—C9	111.99 (10)	C12—C13—H13B	109.6
N2—C8—H8A	109.2	H13A—C13—H13B	108.1
C9—C8—H8A	109.2	O2—N1—O3	122.35 (11)
N2—C8—H8B	109.2	O2—N1—C3	118.53 (11)
C9—C8—H8B	109.2	O3—N1—C3	119.11 (10)
H8A—C8—H8B	107.9	C7—N2—C8	122.84 (11)
N3—C9—C8	110.22 (10)	C7—N2—H1	117.3 (12)
N3—C9—H9A	109.6	C8—N2—H1	119.8 (12)
C8—C9—H9A	109.6	C9—N3—C10	111.82 (10)
N3—C9—H9B	109.6	C9—N3—C13	112.22 (10)
C8—C9—H9B	109.6	C10—N3—C13	108.56 (11)
H9A—C9—H9B	108.1	C12—O4—C11	109.63 (11)
N3—C10—C11	109.46 (13)		
			
C7—C1—C2—C3	−179.68 (10)	N3—C10—C11—O4	60.36 (17)
C6—C1—C2—C3	0.69 (17)	O4—C12—C13—N3	−57.64 (18)
C1—C2—C3—C4	0.62 (18)	C2—C3—N1—O2	172.71 (11)
C1—C2—C3—N1	−176.92 (10)	C4—C3—N1—O2	−4.89 (17)
C2—C3—C4—C5	−0.61 (18)	C2—C3—N1—O3	−6.09 (17)
N1—C3—C4—C5	176.93 (11)	C4—C3—N1—O3	176.31 (11)
C3—C4—C5—C6	−0.7 (2)	C1—C7—N2—C8	−178.52 (11)
C4—C5—C6—O1	−178.16 (12)	C9—C8—N2—C7	−106.90 (14)
C4—C5—C6—C1	1.93 (19)	C8—C9—N3—C10	−163.51 (11)
C2—C1—C6—O1	178.21 (11)	C8—C9—N3—C13	74.22 (14)
C7—C1—C6—O1	−1.41 (18)	C11—C10—N3—C9	177.47 (12)
C2—C1—C6—C5	−1.89 (17)	C11—C10—N3—C13	−58.19 (15)
C7—C1—C6—C5	178.49 (11)	C12—C13—N3—C9	−178.84 (12)
C2—C1—C7—N2	179.62 (11)	C12—C13—N3—C10	57.05 (16)
C6—C1—C7—N2	−0.75 (18)	C13—C12—O4—C11	57.82 (18)
N2—C8—C9—N3	174.64 (11)	C10—C11—O4—C12	−59.34 (18)

### Hydrogen-bond geometry (Å, °)

**Table:**

*D*—H···*A*	*D*—H	H···*A*	*D*···*A*	*D*—H···*A*
N2—H1···O1	0.89 (2)	1.99 (2)	2.6760 (14)	133 (2)
N2—H1···O1^i^	0.89 (2)	2.24 (2)	2.9587 (14)	138 (2)
C4—H4···O4^ii^	0.93	2.47	3.3547 (16)	160
C7—H7···O3^iii^	0.93	2.43	3.3020 (15)	157
C13—H13B···Cg2^iv^	0.97	2.99	3.9254	162

Symmetry codes: (i) −*x*, −*y*+1, −*z*+1; (ii) *x*, *y*+1, *z*+1; (iii) −*x*+2, −*y*+2, −*z*+1; (iv) −*x*+1, −*y*+1, −*z*+1.
